# The Wick Sign After Cold Snare Polypectomy: A Case Report

**DOI:** 10.7759/cureus.8073

**Published:** 2020-05-12

**Authors:** Gregory T Brennan, Gregory Albers

**Affiliations:** 1 Gastroenterology, Texas Digestive Disease Consultants, Dallas, USA; 2 Gastroenterology, University of California Irvine Health, Orange, USA

**Keywords:** colonoscopy complications, polypectomy, colonoscopy, gastroenterology

## Abstract

Cold snare polypectomy is now the preferred technique for resection of most small colorectal polyps. Endoscopists should be aware of a particular mucosal defect after cold snare polypectomy called the wick sign. The wick sign does not represent residual polyp and is not associated with adverse outcomes. Accurate identification is important to prevent unnecessary intervention. A case is presented here with characteristic findings after polypectomy.

## Introduction

Cold snare polypectomy is the preferred method for resection of diminutive or small colorectal polyps as recommended by new guidelines [1]. Following resection, the ability to accurately inspect and characterize the defect is important. The wick sign is a central protrusion in the mucosal defect after cold snare polypectomy. Here we present a typical case with the characteristic endoscopic findings and discuss its management.

## Case presentation

**Figure 1:**
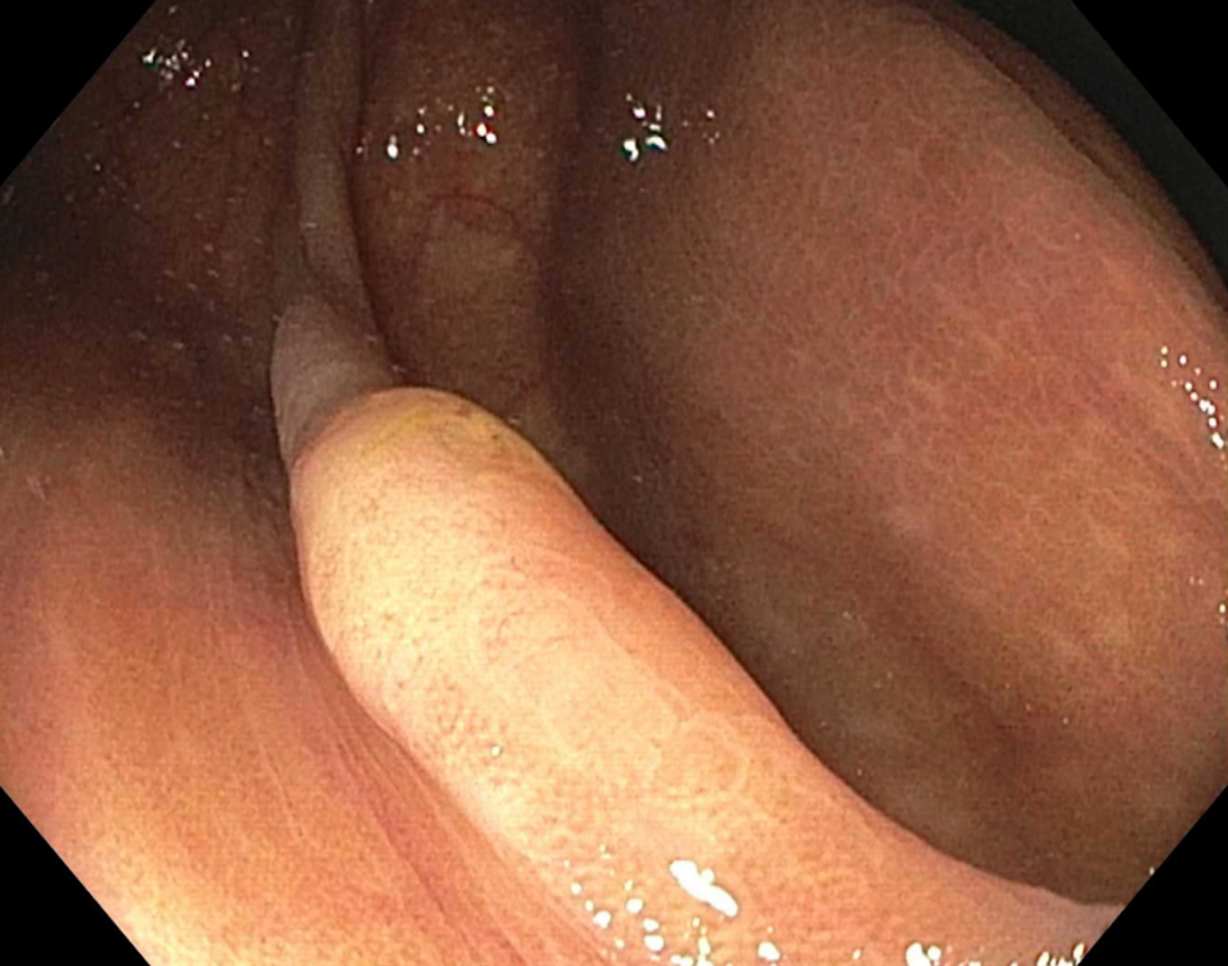
Polyp prior to cold snare resection

A 51-year-old woman presented for screening colonoscopy. She did not have a family history of colorectal cancer and this was her first colonoscopy. In the ascending colon, there was a pale-colored, 6-mm sessile polyp with a mucus cap and indistinct borders seen under high-definition white light (Figure 1).

**Figure 2:**
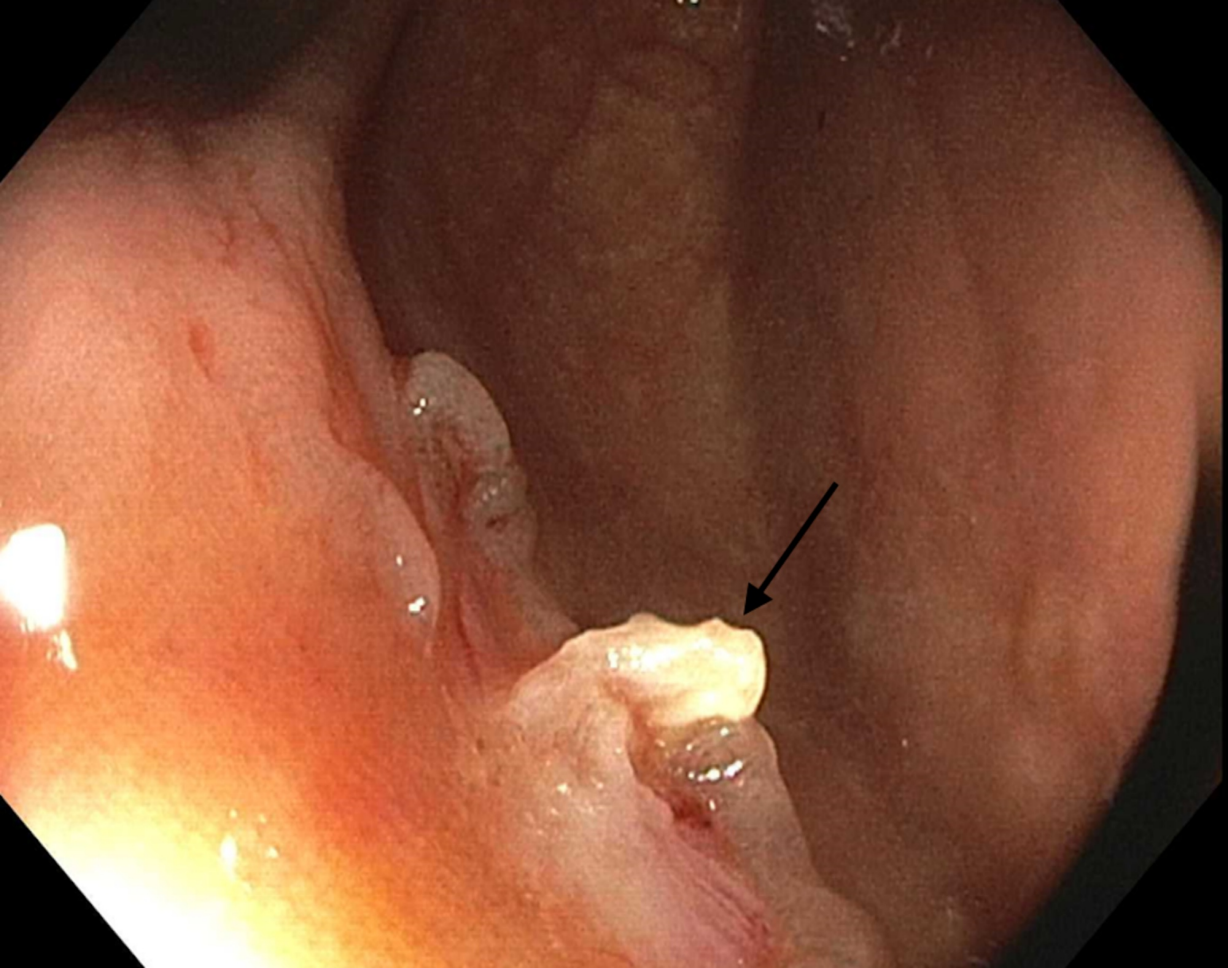
Wick sign (arrow) following cold snare polypectomy

Cold snare polypectomy [using a 10-mm Boston Scientific cold snare (Boston Scientific, Marlborough, MA)] was performed. There was a central protrusion in the polypectomy defect, called the wick sign (Figure 2).

A careful inspection of the defect revealed no evidence of submucosal injury (target sign) or residual polyp. No biopsies were taken from the central protrusion. The patient did well without complication and was discharged home. Final pathology revealed a sessile serrated adenoma.

## Discussion

Cold snare polypectomy is the preferred technique for resection of diminutive and small colorectal polyps. Following polypectomy, a protrusion or cord of white tissue in the center of the mucosal defect is sometimes seen. The shape and color of the protrusion resemble a candle wick with a flame-like appearance, and it is referred to as the wick sign. This finding has also been called a submucosal cord or protrusion. In contrast, the target sign is a deeper depression of concentric rings with visible submucosal fat consistent with a perforation.

In a prospective observational study of 88 consecutive patients undergoing cold snare polypectomy of 257 polyps, Tutticci et al. described the frequency, predictors, and histologic constituents of these central protrusions, which they called cold snare defect protrusions (CSDPs) [2]. They found that CSDPs (wick sign) occurred in 36 (14%) of polypectomy defects. CSDPs were associated with polyp size of 6 mm or larger but were not associated with polyp location, histology, or shape. Histopathologic examination of CSDPs revealed submucosa in 34 (94%) and muscularis mucosa in 29 (80%). The most important finding from the study was that no residual adenomatous or serrated polyp tissue was detected.

It is believed that this protrusion is formed when there is a significant mechanical force applied to the snare [3]. It is sometimes necessary to pull the snare into the colonoscope working channel to achieve a stronger cutting force on the polyp. This explains why the wick sign is seen in polyps >6 mm, as these lesions require greater mechanical force to cut. Tutticci et al. showed that despite greater mechanical force, there was no increase in adverse events [2].

## Conclusions

It is important for gastroenterologists to recognize this finding because the wick sign does not represent residual polyp or a visible vessel. It is not associated with adverse outcomes. The available literature suggests that there is no need for further biopsy, ablation, clipping, or closure of the defect.
